# Induced Regulatory T Cells Attenuate Poly I:C‐Triggered Acute Lung Injury by Modulating Cytokine Responses

**DOI:** 10.1155/jimr/4010824

**Published:** 2026-05-14

**Authors:** Chun-Hsien Hsu, Win-Chin Chiang, Shau-Kwaun Chen, Yung-Feng Lin, Kai-Lee Wang

**Affiliations:** ^1^ Department of Family Medicine, Taipei City Hospital, Heping Fuyou Branch, Taipei, Taiwan, tch.gov.taipei; ^2^ Wanhua Outpatient Department, Taipei City Hospital, Heping Fuyou Branch, Taipei, Taiwan, tch.gov.taipei; ^3^ School of Medicine, College of Medicine, Fu Jen Catholic University, Taipei, Taiwan, fju.edu.tw; ^4^ Department of Exercise and Health Sciences, University of Taipei, Taipei, Taiwan, utaipei.edu.tw; ^5^ Department of Family Medicine, Shin Kong Wu Ho-Su Memorial Hospital, Taipei, Taiwan, skh.org.tw; ^6^ Department of Mechanical Engineering, College of Engineering, National Yang Ming Chiao Tung University, Hsinchu, Taiwan, nctu.edu.tw; ^7^ Jowin Biopharma Inc, Taipei, Taiwan; ^8^ Institute of Neuroscience, National ChengChi University, Taipei, Taiwan, nccu.edu.tw; ^9^ School of Medical Laboratory Science and Biotechnology, College of Medical Science and Technology, Taipei Medical University, Taipei, Taiwan, tmu.edu.tw; ^10^ Department of Long-Term Care, College of Nursing, Asia University, Taichung, Taiwan, asia.edu.tw

**Keywords:** acute lung injury, inflammation, poly I:C, regulatory T cells

## Abstract

**Background and Objective:**

Acute lung injury (ALI) is characterized by severe pulmonary inflammation and edema and carries a high risk of mortality. This study aimed to evaluate the therapeutic potential of induced regulatory T cells (iTregs) as an early intervention to mitigate ALI using a polyinosinic‐polycytidylic acid (poly I:C)‐induced murine model.

**Methods:**

C57BL/6 mice were intratracheally administered the synthetic double‐stranded RNA (dsRNA) analog poly I:C to induce a hyperinflammatory response, followed by intravenous injection of iTreg 1 h later to target the early phase of injury progression.

**Results:**

Analysis of bronchoalveolar lavage fluid (BALF) from poly I:C‐treated mice revealed significantly elevated proinflammatory cytokines (interleukin [IL])‐6, IL‐12, TNF‐α, interferon [IFN]‐β, and IP‐10), decreased anti‐inflammatory cytokines (IL‐10 and transforming growth factor [TGF‐β]), increased infiltration of neutrophils, monocytes, and lymphocytes, and evident alveolar damage with thickened walls and edema. Early iTreg administration effectively reversed these effects by suppressing proinflammatory cytokines, restoring IL‐10 levels, reducing immune cell infiltration, and mitigating tissue damage.

**Conclusions:**

These findings demonstrate that iTreg therapy effectively modulates the hyperinflammatory response in ALI and may represent a promising strategy for treating severe inflammatory lung diseases.

**Summary**

• Induced regulatory T cells (iTregs) administration mitigates polyinosinic‐polycytidylic acid (poly I:C)‐induced acute lung injury (ALI) by reducing immune cell infiltration.
• iTregs suppress pro‐inflammatory cytokines and restore anti‐inflammatory cytokines in bronchoalveolar lavage fluid (BALF).
• Early iTregs therapy alleviates alveolar damage and pulmonary edema in a murine ALI model.
• iTregs Mitigate poly I:C‐Induced ALI.

## 1. Introduction

Pneumonia caused by respiratory viruses, such as coronavirus, influenza virus, rhinovirus, adenovirus, and respiratory syncytial virus, may lead to severe complications and deaths, most of which are secondary to acute lung injury (ALI) and acute respiratory distress syndrome (ARDS) [1]. ARDS is a life‐threatening condition characterized by severe hypoxemia and diffuse lung inflammation. Options for supportive management, such as mechanical ventilation, extracorporeal membrane oxygenation, and glucocorticoids, are limited by high cost and associated complications [2]. The clinical efficacy of corticosteroids in treating ARDS remains controversial. For example, one study has shown that early corticosteroid treatment in COVID‐19‐induced ARDS may increase the risk of ventilator‐associated pneumonia, leading to higher mortality rates within 90 days of administration [3], but corticosteroid use may delay viral clearance [4]. These highlight the ongoing debate on the benefits of steroid therapy for ARDS. Moreover, the high ARDS mortality rate despite existing therapies emphasizes the urgent need for more effective treatments [5, 6]. Polyinosinic‐polycytidylic acid (poly I:C), a synthetic double‐stranded RNA (dsRNA), is widely used to mimic.

A poly I:C is a synthetic dsRNA analog with an average size ranging from 1.5 to 8 kb. Structurally resembling viral dsRNA, it is widely used as an immune stimulant to mimic viral‐induced lung injury in preclinical studies, providing a reproducible model for early inflammatory responses in ARDS [7, 8]. Poly I:C activates innate immune responses by triggering toll‐like receptor 3 signaling pathway, thereby, stimulating the production of proinflammatory cytokines and chemokines, such as tumor necrosis factor‐α (TNF‐α), interleukin (IL) 6, IP‐10, and type I interferons (IFNs) α and β [9]. IFN‐α/β exerts broad immunomodulatory effects, including natural killer cell activation and regulation of humoral immunity [10]. Owing to its potent immunostimulatory properties, poly I:C is extensively used in preclinical research on antiviral immunity, inflammatory disease mechanisms, and hyperinflammatory responses associated with viral infections [11, 12].

Understanding the causes of ARDS deaths is crucial for developing effective treatment strategies. The most severe manifestations of ARDS include alveolar damage, pulmonary edema, and dysregulated immune responses, including excessive neutrophil infiltration and overproduction of proinflammatory cytokines, known as a cytokine storm [13]. In ARDS, alveolar macrophages play critical roles in inflammation, regulated cell death, immune modulation, and tissue repair, influencing both disease progression and resolution. Consequently, regulating the function of alveolar macrophages has been an effective strategy to improve ARDS [14]. Recovery from lung injury depends on the coordinated interactions among neutrophils, macrophages, and lymphocytes, as well as a tightly regulated cytokine signaling. A small proportion of patients with COVID‐19 infected was reported to develop immune dysregulation and cytokine storm, which was marked by T cell lymphopenia and elevated serum levels of IL‐6, IL‐10, and TNF‐α, leading to critical illness and death [15]. When lymphocyte levels decreased, with >14 neutrophil‐to‐lymphocyte ratio, ARDS prognosis significantly worsened [16, 17]. Severe COVID‐19 reportedly leads to lung injury through Dickkopf‐1‐mediated dysregulation of CD4^+^ and CD8^+^ T cells, resulting in reduced antiviral responses and enhanced tissue injury [18].

The pathogenesis of ARDS involves a complex interplay of inflammatory mediators that amplify lung injury; its onset is often triggered by an inflammatory response, characterized by fluid accumulation and activation of neutrophils, which are the first line of defense in the innate immune response. Neutrophil activation leads to excessive production of reactive oxygen species, reactive nitrogen species, and neutrophil extracellular traps, which result in significant alveolar cell damage and increased secretion of proinflammatory cytokines [19], such as TNF‐α, IL‐1, and IL‐6, which play crucial roles in driving cytokine storm and immune imbalance in patients with ARDS. This cascade results in increased oxidative stress, activation of inflammatory pathways, and lung injury exacerbation, which collectively contribute significantly to ARDS progression [20]. Alternatively, antiinflammatory cytokines, such as IL‐10 and transforming growth factor (TGF)‐β, are notably reduced in ARDS. TGF‐β can reduce alveolar space fluid retention and prevent pulmonary edema by stimulating the sodium channels in alveolar epithelial cells [21]. Therefore, decreased TGF‐β in ARDS will lead to pulmonary edema. Disruption in the balance between proinflammatory and antiinflammatory factors is a key feature of ARDS pathophysiology [13, 22]. Targeting the inflammatory response using inhibitors of IL‐1 and IL‐6 has emerged as a potential strategy for managing ARDS caused by COVID‐19 [23]. These early innate immune events contribute to immune dysregulation, highlighting the potential role of regulatory T cells (Tregs) in restoring immune homeostasis [24].

Tregs comprise a T cell subgroup that expresses specific surface markers, including CD4, CD25, and the transcription factor Foxp3, and play an essential role in maintaining immune homeostasis by regulating immune activation and promoting tissue repair through the production of antiinflammatory cytokines, such as IL‐10 and TGF‐β. Building on these early innate immune events [12, 24–29], augmenting the number or function of Tregs may offer therapeutic potential in mitigating lung injury and controlling immune dysregulation in ARDS [16]. Enhancing mitochondrial function in Tregs can also promote their differentiation and potentially improve ARDS outcomes [11, 16]. However, in ARDS, Tregs are often reduced in number and function, leading to uncontrolled inflammation. Induced Tregs (iTregs) generated ex vivo under defined conditions have been shown to effectively suppress inflammation and facilitate tissue repair in preclinical models of inflammatory diseases. However, their therapeutic applications in ARDS remain underexplored.

The study aimed to evaluate the efficacy of iTregs in a poly I:C‐induced ARDS murine model, which mimicked the hyperinflammatory response in viral infections. By assessing histopathological changes, cytokine profiles, and inflammatory cell infiltration, we elucidated the potential of iTreg therapy as a novel approach in ARDS. These findings may provide valuable insights into the translational potential of iTreg‐based therapies in clinical settings, particularly for managing severe inflammatory lung diseases, such as ARDS.

## 2. Materials and Methods

### 2.1. Animal Model and Treatment

Female C57BL/6 mice aged 10 weeks were used to establish an ALI model reflecting the key pathological features of ARDS, in accordance with the American Thoracic Society (ATS) recommendations. This sex was selected based on prior studies demonstrating reduced variability and more stable inflammatory responses in female ALI models [30]. To ensure experimental consistency and reproducibility, each experiment was independently repeated at least three times with at least six mice per group, yielding consistent results. The mice were randomly assigned into three groups (*n* = 6 per group). The control group received phosphate‐buffered saline (PBS) as negative control. The ALI group was intratracheally administered 50‐µg poly I:C, followed by an intravenous injection of PBS (vehicle‐only treatment). The ALI + iTreg group received intratracheal 50‐µg poly I:C, followed by intravenous iTregs (1 × 10^7^) after 1 h to allow initiation of the inflammatory cascade and intervene during the acute phase of lung injury. This dose was selected based on our pilot testing, and no adverse reactions were observed in any mice following the tail‐vein injection.

All animals were housed in specific pathogen‐free conditions, with food and water provided ad libitum, and monitored daily for clinical signs of distress or behavioral changes, such as labored breathing and lethargy. The animal procedures were approved by the Institutional Animal Care and Use Committee under Protocol Number P11203.

To assess lung injury in accordance with the ATS guidelines, we evaluated key features of ALI, including inflammation (i.e., cellular infiltration in bronchoalveolar lavage fluid [BALF]), alveolar–capillary barrier disruption, and lung edema. Lung tissue sections were histopathologically analyzed for structural damage and inflammatory cell infiltration. These assessments allowed comprehensive evaluation of injury severity and the therapeutic effects of iTregs.

### 2.2. Treg Cell Isolation and Induction

Naïve CD4^+^ T cells were isolated from C57BL/6 mice spleens and enriched using the MagniSort Mouse CD4 Naïve T Cell Enrichment Kit (Thermo Fisher Scientific, USA) [31]. Enriched cells were cultured in RPMI‐1640 medium supplemented with 10% FBS, 1% penicillin–streptomycin, 50‐μM β‐mercaptoethanol (2‐ME), and 2‐mM L‐glutamine. For iTregs differentiation, cells were activated with antiCD3/CD28 Dynabeads (Thermo Fisher Scientific, USA) in the presence of 2‐ME (50 μM), IL‐2 (100 U/mL), TGF‐β1 (5 ng/mL), and all‐trans retinoic acid (100 nM).

After 5 days of culture, the cells were harvested and assessed for purity by flow cytometry using the True‐Nuclear Transcription Factor Buffer Set (BioLegend, San Diego, CA, USA). The following fluorochrome‐conjugated antibodies were used: anti‐mouse CD4‐FITC (Cat. No. 100510, BioLegend); anti‐mouse CD25‐PE (Cat. No. 102008, BioLegend); and anti‐mouse Foxp3‐APC (Cat. No. 17‐5773‐82, Thermo Fisher Scientific/eBioscience). Flow cytometry was performed to identify and quantify iTreg populations. The gating strategy was established to ensure consistency, where doublets were excluded and lymphocytes were gated based on size and granularity. CD4^+^, CD25^+^, and Foxp3^+^ populations were sequentially identified. To ensure experimental consistency, iTreg identity and purity were verified prior to each adoptive transfer across three independent batches. The representative gating strategy and iTreg identity/purity (>97% CD4^+^ CD25^+^ Foxp3+) were verified by flow cytometry prior to adoptive transfer (as detailed in Figure S1). The resulting iTregs were washed and resuspended in PBS at a concentration of 1 × 10^7^ cells/0.1 mL/mouse for intravenous injection into the ALI model mice.

### 2.3. Tissue Collection and Histopathological Analysis

On day 2 (~48 h) after iTreg administration, mice were euthanized, and lung tissues were collected for BALF analysis and histopathological examination. One lung was lavaged with 1‐mL PBS to obtain BALF, which was centrifuged (1200 rpm, 4°C, 10 min) to separate the supernatant and cell pellet. The cell pellet was processed in a ProCyte hematology analyzer (IDEXX Laboratories, Westbrook, ME, USA) to assess populations of immune cells, including white blood cells (WBCs), neutrophils, monocytes, and lymphocytes; whereas the supernatant was used for total protein quantification using Bradford assay and cytokine analysis. The other lung was fixed in 10% neutral‐buffered formalin and later processed for histopathological evaluation. To ensure an unbiased evaluation, histopathological assessments were performed in a blinded manner. Lung injury severity was quantified using a standardized scoring system based on the ATS workshop report [32], evaluating criteria such as alveolar congestion, hemorrhage, leukocyte infiltration, and alveolar wall thickness. Quantitative analysis of the stained areas was performed using ImageJ software (National Institutes of Health, USA).

### 2.4. Hematoxylin and Eosin Staining

To assess tissue morphology, alveolar damage, and inflammatory changes, formalin‐fixed, paraffin‐embedded lung tissues were sectioned at 4‐μm thickness, deparaffinized in xylene, and rehydrated through graded ethanol. Sections were stained with hematoxylin for nuclear visualization and eosin for cytoplasmic and extracellular matrix staining. After dehydration and clearing, sections were mounted with coverslips. A veterinary pathologist who was blinded to the treatment groups performed histopathological examination, focusing on alveolar structure, immune cell infiltration, and edema.

### 2.5. Cytokine Detection

Cytokine levels in BALF were quantified using the following respective enzyme‐linked immunosorbent assay (ELISA) kits, according to the manufacturers’ protocols and as previously described in murine ALI/ARDS models [33]: mouse IL‐6 (Cat. No. AB222503, Abcam, Cambridge, UK) [34]; mouse IL‐10 (Cat. No. RK00016, ABclonal, Woburn, MA, USA); mouse TNF‐α (Cat. No. 88‐7324, Thermo Fisher Scientific, Waltham, MA, USA); LEGEND MAX mouse IFN‐β (Cat. No. 439407, Biolegend, San Diego, CA, USA); LEGEND MAX mouse TGF‐β1 (Cat. No. 436707, Biolegend, San Diego, CA, USA); LEGEND MAX mouse IL‐12 (Cat. No. 433607, Biolegend, San Diego, CA, USA); and mouse IP‐10 (Cat. No. BMS6018, Invitrogen, Waltham, MA, USA). Briefly, BALF samples were diluted as needed and incubated in precoated plates with the appropriate capture antibodies. After washing, we added detection antibodies, followed by enzyme conjugate and substrate. Absorbance was measured at 450 nm using a TECAN Infinite 200 PRO microplate reader (Tecan, Männedorf, Switzerland). To account for variations in sample dilution and recovery, the total protein concentration in the BALF supernatant was quantified using a Bradford protein assay (Bio‐Rad Laboratories, Hercules, CA, USA). Cytokine concentrations were normalized to the total protein content and expressed as ug/mg after protein‐based normalization. All measurements were performed in duplicate to ensure reproducibility and minimize batch effects.

### 2.6. Lung Injury Scoring

Lung injury severity was quantified using a standardized scoring system based on the ATS) workshop report [32]. Histopathological parameters, including neutrophil infiltration (alveolar and interstitial), hyaline membrane formation, proteinaceous debris, and alveolar septal thickening, were assessed across multiple high‐power fields (400× total magnification). Each parameter was scored according to severity, and a weighted composite lung injury score (LIS) was calculated using the following formula:

LIS=20×A+14×B+7×C+7×D+2×E/number of fields.

ALI scoring system.

**Table:** 

Item	Parameter	0	1	2
A	Neutrophils in alveolar space	None	1–5	>5
B	Neutrophils in interstitial space	None	1–5	>5
C	Hyaline membrane	None	1	>1
D	Proteinaceous debris in airspace	None	1	>1
E	Alveolar septal thickening	<2X normal	2X–4X	>4X normal

*Note:* Each parameter was scored from 0 to 2, with higher scores indicating more severe acute lung injury (ALI). Total ALI score = sum of all parameters (range 0–10).

### 2.7. Terminal Deoxynucleotidyl Transferase‐Mediated dUTP Nick End Labeling (TUNEL) Assay for Apoptosis Detection

TUNEL assay was performed to detect apoptotic cells in lung tissue sections from the three experimental groups using the in situ cell death detection kit (Cat. No. 11684817910, Roche, Basel, Switzerland), following the manufacturer’s instructions. Briefly, 4‐μm lung tissue sections were subjected to proteinase K digestion and blockade of endogenous peroxidase activity with 3% hydrogen peroxide. The sections were then incubated with the TUNEL reaction mixture at 37°C for 60 min in a humidified chamber. The percentage of apoptotic cells, which were labeled red by TUNEL staining, was calculated and expressed as mean ± standard deviation (SD) for each group.

### 2.8. Statistical Analysis

Data were presented as mean ±SD. For each experiment, a minimum of six mice per group were used, unless otherwise specified. Each experiment was repeated independently at least twice to confirm reproducibility. Statistical analyses were performed using SPSS software (IBM Corp., Armonk, NY, USA). Comparisons between groups were conducted using an unpaired Student’s *t*‐test, whereas multiple‐group comparisons were analyzed by one‐way analysis of variance, followed by Tukey’s post hoc test. A *p*‐value of <0.05 was considered statistically significant.

## 3. Results

### 3.1. iTregs Reduce Inflammatory Cell Infiltration and Cytokine Imbalance in BALF

The WBC, neutrophil, lymphocyte, and monocyte counts were significantly higher in the ALI group than in the control group (*p* < 0.01) but were significantly lower in the ALI + iTreg group than in the ALI group (*p* < 0.05) (Figure 1). The levels of total protein and proinflammatory cytokines, including IL‐6, IL‐12, TNF‐α, IFN‐β, and IP‐10, were significantly higher in the ALI group than in the control group (*p* < 0.01); the levels of these inflammatory markers were significantly attenuated in the ALI + iTreg group (*p* < 0.05) (Figure 2). For the anti‐inflammatory cytokine levels in BALF using the control group for comparison, IL‐10 was lower in the ALI group but higher in the ALI + iTreg group, whereas TGF‐β1 was higher in the ALI group and was increased even higher in the ALI + iTreg group (Figure 2).

Figure 1Immune cell infiltration in bronchoalveolar lavage fluid. Levels of WBCs (A), neutrophils (B), lymphocytes (C), and monocytes (D) in the control, ALI, and ALI + iTreg groups (*n* = 6 for each group). Compared with the control group, the ALI group has significantly higher cell counts, whereas the ALI + iTreg group shows lower cell counts. Data are presented as mean ± SEM. Statistical significance was determined by one‐way ANOVA followed by Tukey’s post hoc test.  ^∗^ *p* < 0.05,  ^∗∗^ *p* < 0.01 vs. control; ^#^ *p* < 0.05 vs. ALI group. Data are representative of at least three independent experiments with similar results.

**Figure:**
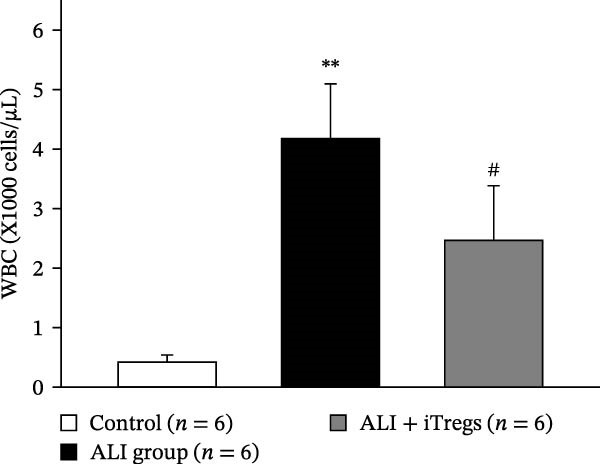
(A)

**Figure:**
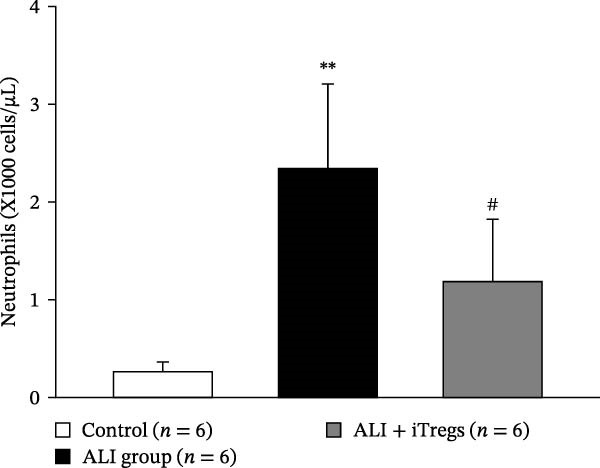
(B)

**Figure:**
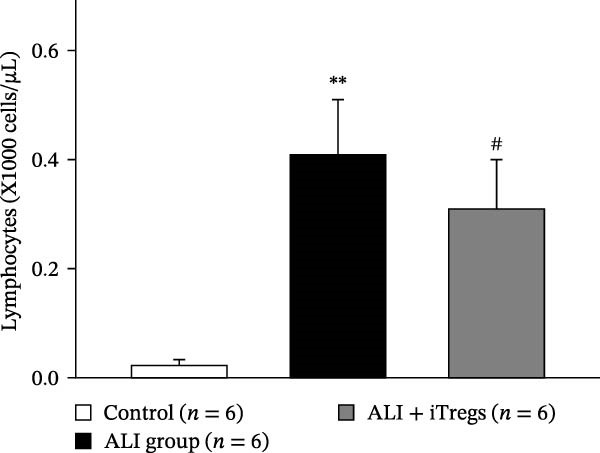
(C)

**Figure:**
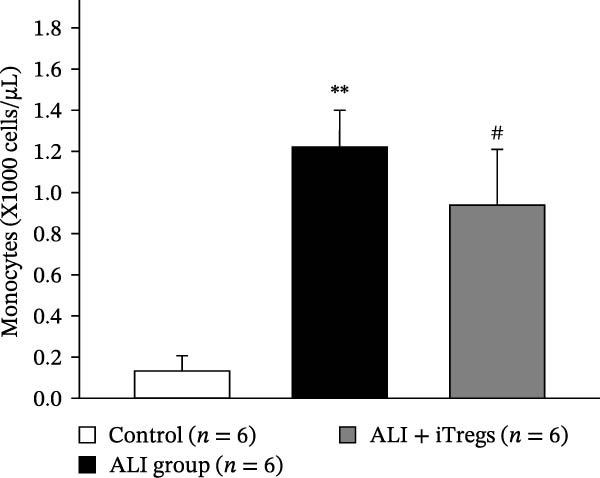
(D)

Figure 2Cytokine levels in BALF in the three groups. Comparisons of the concentrations of total protein (A), IL‐6 (B), IL‐12 (C), TNF‐α (D), IFN‐β (E), IP‐10 (F), IL‐10 (G), and TGF‐β1 (H) in BALF among the three groups are shown. Sample sizes (*n* = 6 per group) are indicated on each panel. These findings demonstrate that iTreg treatment significantly modulates inflammatory and antiinflammatory cytokine profiles, effectively mitigating the inflammatory response in the ALI murine model. BALF, bronchoalveolar lavage fluid; UD, undetectable. Data are presented as mean ± SD. Statistical significance was determined by one‐way ANOVA followed by Tukey’s post hoc test. *p < 0.05 and **p < 0.01, ALI group vs. control group; ^#^ *p*  < 0.05, ALI + iTreg group vs. ALI group. Data are representative of at least three independent experiments with similar results.

**Figure:**
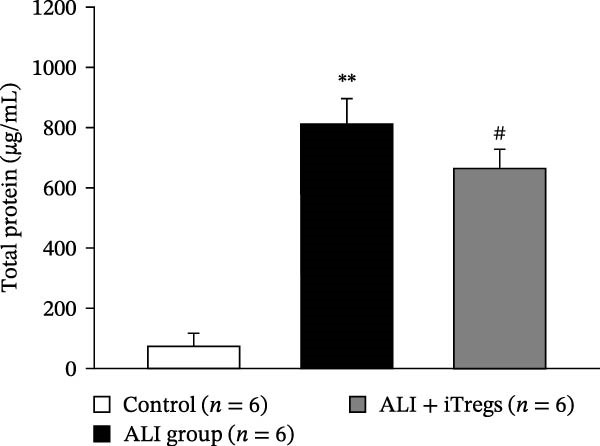
(A)

**Figure:**
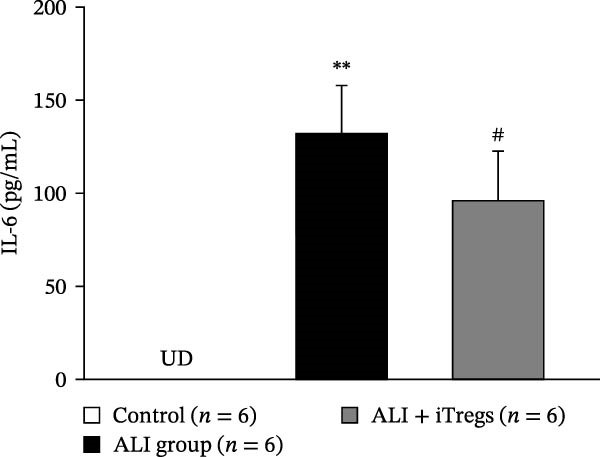
(B)

**Figure:**
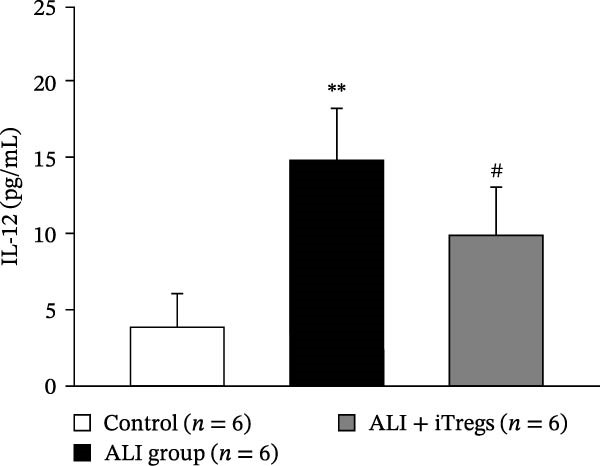
(C)

**Figure:**
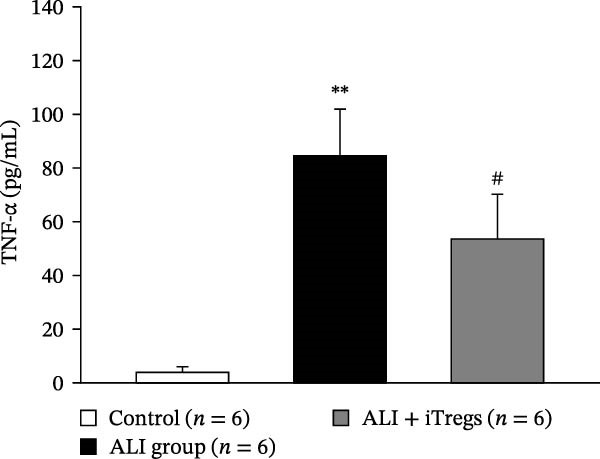
(D)

**Figure:**
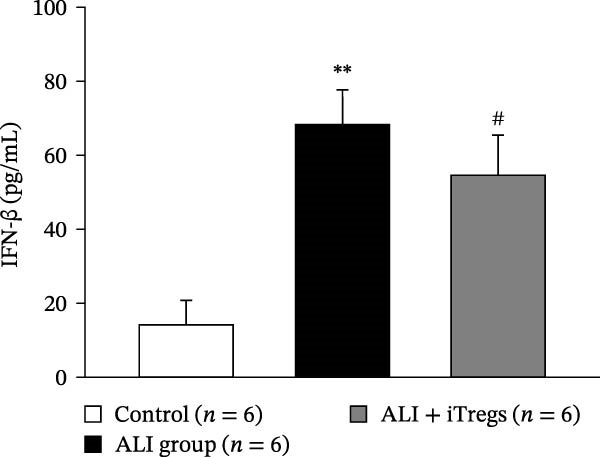
(E)

**Figure:**
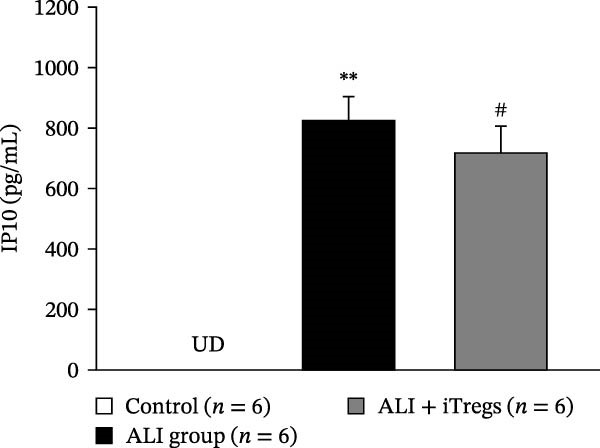
(F)

**Figure:**
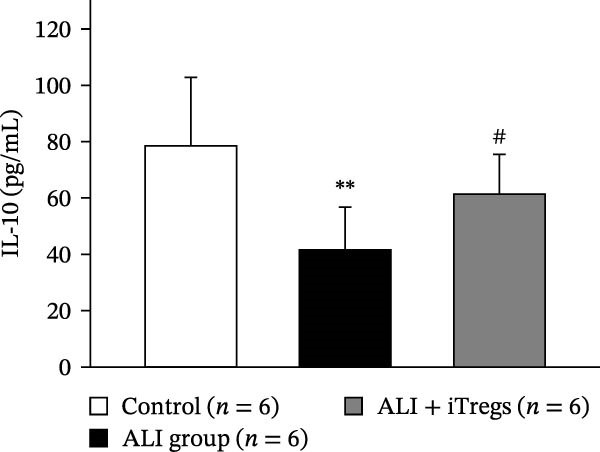
(G)

**Figure:**
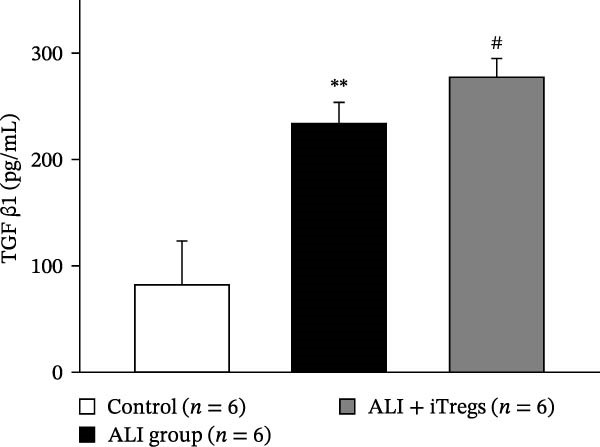
(H)

### 3.2. iTregs Alleviate Lung Tissue Damage and Reduce Histological Injury Scores

**Figure 3:**
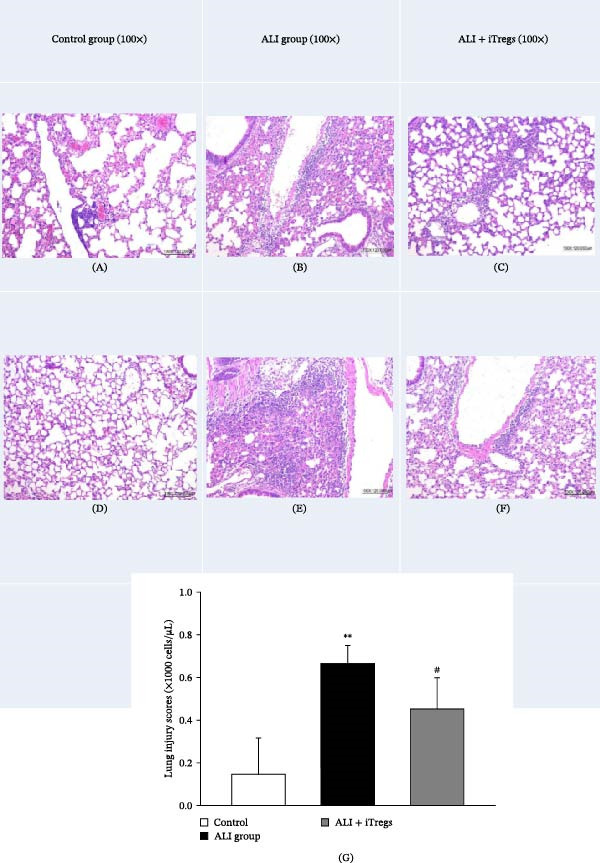
Representative lung sections and corresponding lung injury scores in each group. (A–F) Representative lung sections from the (A, D) control; (B, E) ALI; and (C, F) ALI + iTreg groups. (G) Quantitative lung injury scores show minimal inflammatory cell infiltration and normal alveolar structure in the control group; significant neutrophil infiltration, alveolar septal thickening, and proteinaceous debris in the ALI group; and reduced inflammatory cell infiltration and improved alveolar structure in the ALI + iTreg group (hematoxylin and eosin stain, 100× magnification). Data are presented as mean ± SD (*n* = 6 per group). Statistical significance was determined by one‐way ANOVA followed by Tukey’s post hoc test.  ^∗∗^ *p* < 0.01 vs. control, ^#^ *p* < 0.05 vs. ALI. Representative images are shown, which were consistent with the results of two independent biological replicates.

In the control group, lung tissues displayed minimal inflammatory cell infiltration and no structural damage. On the other hand, the ALI group exhibited marked neutrophil infiltration in the alveolar and interstitial spaces, along with significant alveolar septal thickening and proteinaceous debris and a significantly higher LIS, compared with that in the control group (*p* < 0.01, Figure 3). In the ALI + iTreg group, iTreg treatment significantly alleviated the histological signs of lung injury, with reduced neutrophil infiltration and alveolar thickening, compared with those in the ALI group. The LIS was significantly lower in the ALI + iTreg group than in the ALI group (*p* < 0.05, Figure 3G).

### 3.3. iTregs Mitigate Poly I:C‐Induced Apoptosis in Lung Tissues

**Figure 4:**
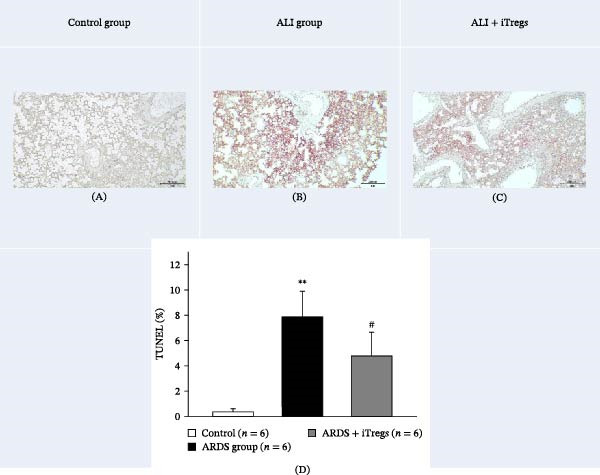
TUNEL assay for evaluating lung tissue apoptosis in the three groups. Representative TUNEL‐stained images show apoptotic cells (red fluorescence) in lung sections from the control (A), ALI (B), and ALI + iTreg (C) groups. (D) Bar graph shows the quantification of apoptotic cells as the percentage of TUNEL‐positive cells per field. Sample sizes (*n*) are indicated on each panel. Data are presented as mean ± SD (*n* = 6 per group). Statistical significance was determined by one‐way ANOVA followed by Tukey’s post hoc test. Scale bars represent 200 μm.  ^∗∗^ *p* < 0.01 vs. control, ^#^ *p* < 0.05 vs. ALI. Representative images are shown, which were consistent with the results of two independent biological replicates.

Representative images of TUNEL‐stained lung sections are shown in Figure 4A, whereas quantification of apoptotic cells is presented in Figure 4B. The percentage of TUNEL‐positive cells was significantly higher in the ALI group than in the control group (7.88% ± 2.01% vs. 0.35% ± 0.26%) but decreased to 4.77% ± 1.89% in the ALI + iTreg group. These findings indicated a protective effect against poly I:C‐induced lung injury and suggested that iTregs mitigate excessive cell death in ALI, potentially contributing to improved lung recovery.

### 3.4. iTregs Modulate High‐Mobility Group Box 1 (HMGB1) Expression, Collagen Deposition, and Treg Localization

**Figure 5:**
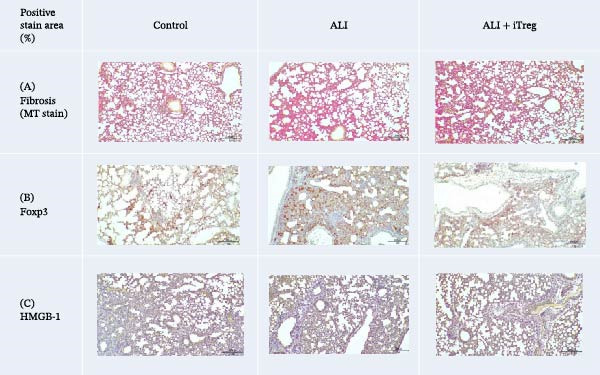
Representative Masson’s trichrome, Foxp3, and HMGB1 staining of lung tissues in the three groups. (A) Masson’s trichrome staining for fibrosis shows increased collagen deposition in the ALI group than in the control group, and this is attenuated by iTreg treatment. (B) Immunohistochemical staining for Foxp3+Tregs demonstrated increased Treg infiltration after iTreg administration. (C) HMGB1 in lung tissue expression is elevated in the ALI group but decreases after iTreg treatment. Scale bars represent 200 μm. Representative images are shown, which were consistent with the results of two independent biological replicates.

To further evaluate the effects of iTreg treatment on lung pathology, we assessed pulmonary inflammation, collagen deposition and fibrosis, and Treg lung localization, usingHMGB1 protein staining, Masson’s trichrome staining, and Foxp3 immunostaining, respectively (Figure 5, Table 1). HMGB1 expression in the lungs was increased in the ALI group (5.60% ± 1.57%) compared to the control group (*p* < 0.01). Following iTreg therapy, HMGB1 levels were significantly attenuated (3.46% ± 1.29%, *p* < 0.05 vs. ALI group), demonstrating the potent anti‐inflammatory effect of the cell therapy. Collagen deposition showed no significant differences among the groups during this acute phase. Regarding Treg localization, Foxp3‐positive areas significantly increased in the ALI group (3.42% ± 0.95%) compared to the control (1.5% ± 0.9%, *p* < 0.01), reflecting a robust endogenous immune response to poly I:C‐induced injury. In the ALI + iTreg group, Foxp3‐positive areas remained elevated (2.65% ± 1.71%) relative to controls. While the total percentage of Foxp3+ area in the treatment group was slightly lower than in the untreated ALI group—potentially due to the overall reduction in total inflammatory cell infiltration as shown by decreased HMGB1 and cytokine levels—it still confirms the presence of regulatory T cells within the injured microenvironment.

**Table 1:** Quantitative analysis of positive staining areas in lung tissue sections.

Positive stain area (%)	Treatment group		
	Control	ALI	ALI + iTreg
Fibrosis (Masson’s trichrome stain)	0.11 ± 0.06	0.08 ± 0.07	0.14 ± 0.07
Foxp3	1.50 ± 0.90	**3.42** ** ± 0.95** ^∗∗^	2.65 ± 1.71
HMGB‐1	2.58 ± 1.05	**5.60** ** ± 1.57** ^∗∗^	**3.46** ** ± 1.29** ^**#**^

*Note:* Data are presented as mean ± SD (*n* = 6 per group). Statistical significance was determined by one‐way ANOVA followed by Tukey’s post hoc test. Bold values indicate statistically significant differences (*p* < 0.05) compared to the other experimental groups.

Abbreviations: ALI, acute lung injury; Foxp3, forkhead box P3; HMGB‐1, high‐mobility group box 1; iTreg, inducible regulatory T cell.

^∗∗^ *p* < 0.01 vs. control.

^#^ *p* < 0.05 vs. ALI.

On the other hand, Foxp3‐positive Tregs were markedly higher in the ALI group than in the control group, and this increase was partially attenuated after iTreg administration. These results suggested that iTregs may contribute to modulating inflammation and supporting tissue conditions conducive to lung repair.

## 4. Discussion

This study successfully established an animal model of ALI induced by poly I:C and demonstrated that iTreg therapy significantly alleviates lung inflammation by reducing pro‐inflammatory cytokines (IL‐6 and TNF‐α) and increasing anti‐inflammatory mediators (IL‐10, TGF‐β). iTregs helped restore immune balance by preventing immune cell overactivation and reducing alveolar epithelial cell death, a critical determinant of lung recovery [35]. Our histopathological analyses confirmed reduced alveolar damage and inflammatory infiltration, highlighting the therapeutic potential of iTregs in viral mimetic ALI.

At the molecular level, poly I:C, as a synthetic analog of viral dsRNA, primarily triggers the TLR3‐mediated cascade. Our findings suggest that iTregs effectively suppress TLR3‐mediated p38 MAPK activation and downregulate NF‐κB signaling, which are central drivers of the “cytokine storm” observed in ALI. Furthermore, iTreg treatment attenuates type I interferon‐related pathways, aligning with the role of Tregs in fine‐tuning the activation threshold of innate immune receptors during viral mimetic challenges. Regarding temporal dynamics, iTreg‐mediated effects appear predominantly acute and transient. Rather than merely delaying the inflammatory peak, iTreg administration shortens the inflammatory phase and facilitates early epithelial repair, expediting the restoration of lung homeostasis.

We utilized poly I:C to model virus‐like ALI due to its ability to activate TLR3‐mediated innate immunity and induce neutrophil infiltration [7, 8]. However, we acknowledge that this synthetic analog does not fully recapitulate bacterial LPS‐mediated or mechanical ventilation‐induced injury; thus, generalizability should be interpreted with caution.

Regarding the distinction between iTregs and natural Tregs (nTregs), our primary aim was to evaluate therapeutic efficacy rather than mechanistic differences. All iTregs were confirmed to be >97% CD4^+^ CD25^+^ Foxp3^+^ (Figure S1). While we did not compare them experimentally, the literature indicates that iTregs and nTregs differ in several biological aspects. Specifically, iTregs are known to exhibit less stable FOXP3 expression in vivo compared with nTregs, which may lead to shorter persistence after adoptive transfer [27, 36, 37]. This difference is often attributed to the incomplete demethylation of the FOXP3 Treg‐specific demethylated region (TSDR) in iTregs. Furthermore, published data indicate that iTregs generally have reduced survival duration in vivo relative to nTregs [36]. These mechanistic characteristics are consistent with the short‐term but clear protective effects observed in our ALI model, where iTregs rapidly resolved acute inflammation but were not expected to provide prolonged immune tolerance.

Regarding the temporal dynamics of this protection, iTreg‐mediated effects in this model appear predominantly acute and transient, acting to accelerate the natural resolution of inflammation. Rather than merely delaying the inflammatory peak, iTreg administration shortens the inflammatory phase and facilitates early epithelial repair, leading to a more rapid restoration of lung homeostasis. While this acute model supports the safety of early intervention, future studies utilizing live pathogen challenges will be essential to fully characterize the long‐term host defense profile. Nonetheless, our model captures the core concept of rapid immune intervention, which is consistent with current clinical efforts to prevent early inflammatory escalation in ALI/ARDS.

The timing of intervention (1 h postchallenge) reflects the therapeutic window in which patients typically receive emergency assessment upon hospital admission. Emerging clinical evidence supports the feasibility of such early interventions; for instance, a phase I clinical trial evaluating Treg therapy for COVID‐19‐associated lung injury (NCT04468971) initiated treatment on hospital day 0, indicating that same day administration is achievable in real‐world settings. This clinical principle is further corroborated by existing literature demonstrating that Treg infusion shortly after injury (e.g., 1 h postchallenge) significantly improves outcomes [38, 39]. Consistent with these findings, our preliminary data showed that pretreatment (1 day before the challenge) was not effective, whereas the postinjury 1 h intravenous approach adopted in this study yielded significant protection. However, we acknowledge that the rapid creation of autologous iTregs poses logistical challenges, as ex vivo expansion typically requires several weeks. To bridge this gap in urgent clinical scenarios, the development of cryopreserved, off the shelf allogeneic products (such as cord blood‐derived Tregs) represents a promising strategy for achieving rapid, same day administration.

Translational considerations must include the risks of allogeneic host versus graft responses and potential‐off target immunosuppressive effects, such as increased susceptibility to opportunistic infections or impaired tumor immunosurveillance. In this study, we utilized a single dose during the acute phase to limit prolonged systemic exposure. To further mitigate these risks in clinical settings, alternative strategies such as localized pulmonary delivery or specialized short‐term treatment protocols could be considered to ensure that the immunosuppressive action remains focused on the site of injury. Furthermore, the development of engineered approaches, including chimeric antigen receptor (CAR) Treg technology, offers a promising avenue for enhancing lung targeted specificity and minimizing systemic side effects.

The maintenance of systemic immune homeostasis in humans can be influenced by various lifestyle and environmental factors [12]. Therefore, moving toward large animal or human trials requires consistent preclinical evidence demonstrating lung‐targeted efficacy with no observable toxicity in extrapulmonary organs. Critical safety parameters include the maintenance of FOXP3 stability to ensure predictable suppressive function and the development of controllable dosing strategies. Current data from large animal transplantation models suggest that specialized Treg products can persist and function without inducing systemic adverse effects, providing a foundation for early phase clinical escalation. However, transitioning to human applications will necessitate rigorous long‐term monitoring for potential risks such as opportunistic infection, tumorigenesis, and broader immune dysfunction. These safety benchmarks are essential for refining iTreg therapy into a viable clinical intervention for ALI (PMC9641266).

Furthermore, considering the clinical safety of such interventions, we addressed whether iTreg‐mediated modulation might increase vulnerability to secondary infections. In our study, iTreg treatment was administered as a single dose during the acute phase rather than chronically. Moreover, since poly I:C is a synthetic mimic and not a replicating virus, no actual pathogen was present to propagate. Our findings demonstrate that iTregs effectively mitigate excessive pulmonary inflammation without evidence of heightened vulnerability. While this acute model supports the safety of early intervention, future studies utilizing live pathogen challenges will be essential to fully characterize the long‐term host defense profile following iTreg therapy.

A key question raised during the review process is whether the observed benefits of iTregs stem from general immunosuppression or precise immunoregulation of lung‐specific pathways. Our findings suggest that the therapeutic effects are more consistent with a selective mitigation of excessive inflammation rather than generalized immune suppression. First, iTreg treatment significantly rebalanced the dysregulated inflammatory environment induced by poly I:C, as evidenced by the marked reduction in BALF total white blood cell, neutrophil, lymphocyte, and monocyte counts (Figure 1), alongside a significant decrease in proinflammatory cytokines such as IL‐6, IL‐12, TNF‐α, IFN‐β, and IP‐10 (Figure 2). Concurrently, the levels of anti‐inflammatory cytokines IL‐10 and TGF‐β1 were significantly increased, reflecting a restoration of immune homeostasis.

Regarding the site of action, our evidence suggests that iTreg activity is primarily localized within the lung tissue rather than mediated through systemic cytokine modulation. This conclusion is supported by the fact that poly I:C was administered directly into the lungs, confining the initial inflammatory challenge to the pulmonary environment. Furthermore, CBC conducted 3 days after induction showed no significant increase in total leukocytes or leukocyte subsets in the disease‐control group compared to healthy controls (data not shown). This lack of systemic inflammatory response indicates that the protective effects of iTreg therapy observed in this study were predominantly exerted within the lung microenvironment, directly targeting localized cytokine cascades such as the IL‐10/TGF‐β axis to resolve alveolar inflammation.

Furthermore, our observations align with established lung‐specific immunoregulatory mechanisms mediated by Tregs in ALI. These include: (1) IL‐10/TGF‐β–dependent reprogramming of macrophages toward an anti‐inflammatory M2 phenotype to promote resolution; (2) regulation of neutrophil dynamics and reduction of chemokines (e.g., CXCL1/2) to prevent alveolar damage; (3) potential amphiregulin‐mediated epithelial repair; (4) activation of lung niche–specific ST2^+^ repair‐Tregs; and (5) endothelial protection to reduce vascular leakage. While we directly measured IL‐10, TGF‐β1, and apoptosis levels—all central components of these pathways—we acknowledge that later‐phase repair markers such as amphiregulin, ST2^+^ expression, and endothelial junction markers were not assessed in this acute‐phase study. We have included this as a limitation in the revised manuscript. Overall, the observed effects of iTregs reflect a targeted attenuation of excessive inflammatory activation, consistent with established lung immunoregulation models, rather than global immunosuppression.

Foxp3+Tregs are essential for maintaining immune homeostasis and resolving inflammation [21]. On the other hand, Treg instability, characterized by diminished Foxp3 expression, has been linked to autoimmune diseases, graft‐versus‐host disease, and cytokine storm, during which Tregs may lose their regulatory phenotype and differentiate into less suppressive or effector‐like cells [40]. Therapeutic approaches such as low‐dose IL‐2 administration aim to enhance Treg populations or improve their stability [26]. These observations align with our findings that Foxp3+Tregs administration markedly improved poly I:C‐induced lung inflammation, highlighting their potential to mitigate hyperinflammatory responses and promote tissue repair.

Inhibitory cytokines, including IL‐10 and TGF‐β, are pivotal mediators of Treg suppressive functions. Elevated IL‐10 levels during the early phase (days 1–3) of severe sepsis have been associated with increased mortality, with nonsurvivors showing higher IL‐10 than survivors [41].

In our study, iTreg administration significantly increased IL‐10 and TGF‐β levels compared with the poly I:C‐treated group. These cytokines were not supplied exogenously, and their elevation likely reflected the immunoregulatory activity of transferred iTregs and/or iTreg‐induced host responses, which correlated with reduced inflammation and cell death. Notably, TGF‐β levels were also elevated in the ALI group, and IFN‐γ levels did not differ significantly between ALI and ALI + iTreg groups (ALI: 336.72, 293.44, 316.77 pg/mL; ALI + iTreg: 324.05, 329.38, 284.44 pg/mL; data not shown), likely due to delayed IFN‐γ expression during later stages of viral‐mimetic inflammation [42]. The short‐term effects of poly I:C may not fully capture IFN‐γ dynamics. These findings underscore the critical regulatory function of inhibitory cytokines in attenuating inflammatory responses.

Tregs can modulate immune responses through secretion of IL‐10, IL‐35, IFN‐γ, and TGF‐β. Notably, Treg‐derived extracellular vesicles provide enhanced stability and safety, representing a promising therapeutic strategy for ARDS and other inflammatory conditions [29]. IL‐10 is primarily secreted by macrophages and Treg cells and is a key antiinflammatory mediator. Compared with the poly I:C‐treated group, our study demonstrated significant increases in IL‐10 and TGF‐β levels following iTreg administration. These cytokines were not supplied exogenously, and their elevation likely reflected the immunoregulatory activity of the transferred iTregs and/or iTreg‐induced host response, which was associated with reduced inflammation and cell death. However, it was noteworthy that TGF‐β levels were also elevated in the ALI group in this study and that there were no significant differences in IFN‐γ levels between the ALI and ALI + iTreg groups (ALI group: 336.72, 293.44, and 316.77 pg/mL vs. ALI + iTreg group: 324.05, 329.38, and 284.44 pg/mL; data not shown). These results were probably due to delayed IFN‐γ expression in the later stages of viral infections, as previously described [42]. The short‐term effects of poly I:C administration in this study may not have fully captured the role and function of IFN‐γ. Nevertheless, these findings underscored the critical regulatory function of inhibitory cytokines in attenuating inflammatory responses and highlighted the therapeutic potential of iTregs in ALI models.

The cytokine IL‐6, which is primarily secreted by activated macrophages and endothelial cells during inflammation, is closely linked with systemic inflammation and septic shock [15, 41, 43, 44] and drives immunometabolic reprogramming, which plays a crucial role in the pathogenesis and severity of viral infection‐associated diseases [45]. IL‐6 elevation has been recognized as a key indicator of disease progression in viral infection‐associated diseases, such as severe COVID‐19 and influenza, often triggering cytokine storm that leads to fatal outcomes [46]. IL‐6 promotes CD4^+^ T cell differentiation into Th17 cells while inhibiting Treg expression, resulting in an imbalance in the Treg/Th17 ratio [46, 47]. This imbalance exacerbates systemic inflammation and delays lung repair, potentially leading to mortality. Our study demonstrated that IL‐6 levels were undetectable in the control group but were significantly elevated in the poly I:C‐treated group (mean ± SD: 132.54 ± 25.26 pg/mL). Notably, reduced IL‐6 levels (mean ± SD: 95.81 ± 26.79 pg/mL) after iTreg treatment correlated with decreased inflammation and apoptosis. This result was consistent with the findings of a previous study [28], which demonstrated that IL‐6 levels were undetectable in healthy controls but were markedly elevated in patients with inflammatory conditions. Moreover, the study highlighted the role of IL‐6 in proinflammatory responses and tissue damage. These findings underscored the immunosuppressive effects of iTregs, which can reduce IL‐6 production and mitigate IL‐6‐mediated inflammatory responses, thereby, offering a promising therapeutic strategy for ALI.

Tregs play a crucial role in mitigating the effects of ARDS through their ability to suppress excessive immune activation and promote tissue repair [48]. As protective factors in various inflammatory and immune‐mediated diseases, Tregs exhibit the capacity to inhibit both innate and adaptive immune cells. In COVID‐19, Tregs play a critical role in limiting excessive cytokine release, particularly by modulating the activities of effector T cells and innate immune cells, such as neutrophils and macrophages. Reduction or dysfunction of Tregs is strongly linked with worsened lung injury and poor prognosis [49].

Globally, several clinical trials have evaluated Treg‐based therapies for ARDS and other inflammatory conditions. Treg therapies have shown a great potential in alleviating symptoms and slowing disease progression in patients with COVID‐19, with approaches combining Tregs with Th2 cells showing promising results in mitigating the severity of organ damage [50, 51]. Moreover, Treg deficiencies impair alveolar epithelial proliferation and delay lung recovery. After injury, Tregs are able to enhance the proliferation of alveolar epithelial type II cells (AT2) and promote vascular remodeling by increasing the secretion of keratinocyte growth factor and vascular endothelial growth factor [25, 52]. The findings of this study, together with previous studies [8, 40] highlighted the multifaceted role of Tregs in controlling inflammation and promoting lung repair through mechanisms such as AT2 proliferation and vascular remodeling. This distinction underscores their therapeutic potential for severe pulmonary injuries.

We acknowledge several main limitations of this study. First, although transferred iTregs were thoroughly washed, a direct effect of residual poly I:C cannot be fully excluded. Second, although Foxp3 staining indicated increased Tregs in the lungs, we did not specifically track transferred versus endogenous cells. Third, iTregs were given shortly after poly I:C instillation, reflecting intervention in the early phase rather than in established ARDS; furthermore, since poly I:C induces inflammation without active viral replication, the protective effects should be interpreted as mitigation of lung injury rather than viral clearance. Fourth, while the identity and high purity (more than 97% CD4+CD25+Foxp3+) of iTregs were consistently verified prior to injection, we acknowledge that additional in‐depth phenotyping and in vitro functional characterization were not performed. Specifically, the expression of supplemental functional markers such as CTLA‐4 and ICOS, as well as TGF‐β‐related markers including LAP and GARP, was not assessed. Furthermore, although previous literature indicates that stable Foxp3+ Tregs typically do not produce pro‐inflammatory cytokines, we did not conduct specific in vitro assays to confirm that our purified iTregs were not producing IFN‐γ, TNF‐α, or IL‐2 prior to adoptive transfer. Importantly, direct in vitro suppression assays, such as CFSE‐based proliferation assays or mixed lymphocyte reactions, were not conducted in this phase of the study. However, the robust in vivo efficacy demonstrated by the significant reduction in inflammatory infiltration, pro‐inflammatory cytokine levels, and LISs collectively supports the potent suppressive activity of the transferred iTregs. Future investigations focusing on the functional stability of these cells under various inflammatory conditions will be essential to further validate these therapeutic mechanisms. Fifth, although our single‐dose tail‐vein administration was effective, testing multiple dosing regimens or alternative delivery routes could further refine the therapeutic window. Sixth, this study did not include transfer groups of naïve CD4^+^ T cells or nTregs, which would have helped distinguish specific iTreg immunomodulation from general Tcell infusion effects. Seventh, while we focused on core pro‐ and anti‐inflammatory cytokines, this study did not evaluate the potential influence of iTreg therapy on other homeostatic axes, such as IL‐2 homeostasis or the Th17 cell balance, nor did it assess interactions with myeloid‐derived suppressor cells (MDSCs). Tregs are known to fine‐tune these systemic immune subsets, and their specific roles in viral‐mimetic ALI warrant further investigation. Furthermore, while iTregs potentially promote epithelial‐protective effects via the restoration of tight junction proteins such as claudin‐5, these molecular markers were not directly assessed in this study. Future investigations focusing on the stabilization of the alveolar‐capillary barrier by iTregs are warranted.

Finally, although our findings and BALF cytokine trends were consistent and reproducible across at least three independent experimental batches, indicating that batch effects were minimal, we utilized only female mice to ensure a stable inflammatory response. This limits the generalizability of our results to both sexes, and future studies should include both male and female mice to investigate potential sex‐based differences in therapeutic efficacy. Despite these limitations, our findings aligned with emerging evidence supporting Treg‐based therapies for severe inflammatory diseases, offering a promising avenue for future translational and clinical applications.

## 5. Conclusion

In conclusion, this study demonstrated that iTreg therapy effectively mitigates poly I:C‐induced ALI by reducing proinflammatory cytokines, enhancing anti‐inflammatory mediators, and attenuating alveolar damage. By optimizing iTreg culture and demonstrating their therapeutic efficacy, our findings provide a strong preclinical foundation for developing Treg‐based interventions in inflammatory lung diseases, highlighting their translational potential for future clinical applications.

## Author Contributions

Study conception, literature search, administration, funding acquisition: Shau‐Kwaun Chen and Yung‐Feng Lin. Study concepts, quality control of data and algorithms: Chun‐Hsien Hsu, Win‐Chin Chiang, and Kai‐Lee Wang. Study design, data acquisition, data analysis and interpretation, statistical analysis: Win‐Chin Chiang. Manuscript preparation and editing: Kai‐Lee Wang. Manuscript review: Chun‐Hsien Hsu and Kai‐Lee Wang.

## Funding

This study was sponsored by the Department of Health, Taipei City Government (Grants 11101‐62‐022, 11201‐62‐033, 11401‐62‐008, and 11501‐62‐011), the Taipei City Hospital (Grants TPCH‐112‐33, TPCH‐113‐30, TPCH‐114‐37, and TPCH‐115‐30), the National Science and Technology Council (Grants NSTC 111‐2314‐B‐532‐001 and 113‐2320‐B‐468‐003), and the Shin Kong Wu Ho‐Su Memorial Hospital (Grants 2020SKHADR021 and 2020SKHADR022), Taiwan (R.O.C.).

## Disclosure

All authors have read and approved the final manuscript. The authors carefully reviewed and revised the content to ensure accuracy and compliance with journal guidelines. The final manuscript reflects the authors’ intellectual contributions, and they take full responsibility for its content.

## Ethics Statement

The animal study protocol was approved by the Institutional Animal Care and Used Committee of Fu Jen Catholic University (Protocol Code P11203).

## Conflicts of Interest

The authors declare no conflicts of interest.

## Supporting Information

Additional supporting information can be found online in the Supporting Information section.

## Supporting information


**Supporting Information** Figure S1: Shows the differentiation process of naïve CD4⁺ T cells into induced regulatory T cells (iTregs) and the analysis of Foxp3 expression by flow cytometry.

## Data Availability

The data that support the findings of this study are available from the corresponding author upon reasonable request.
