# Supplementary figures and images for: Induced Regulatory T Cells Attenuate Poly I:C‐Triggered Acute Lung Injury by Modulating Cytokine Responses
Source: J Immunol Res. 2026 May 14;2026:4010824. doi: 10.1155/jimr/4010824 (PMC13176617; doi:10.1155/jimr/4010824)

## Slide 1
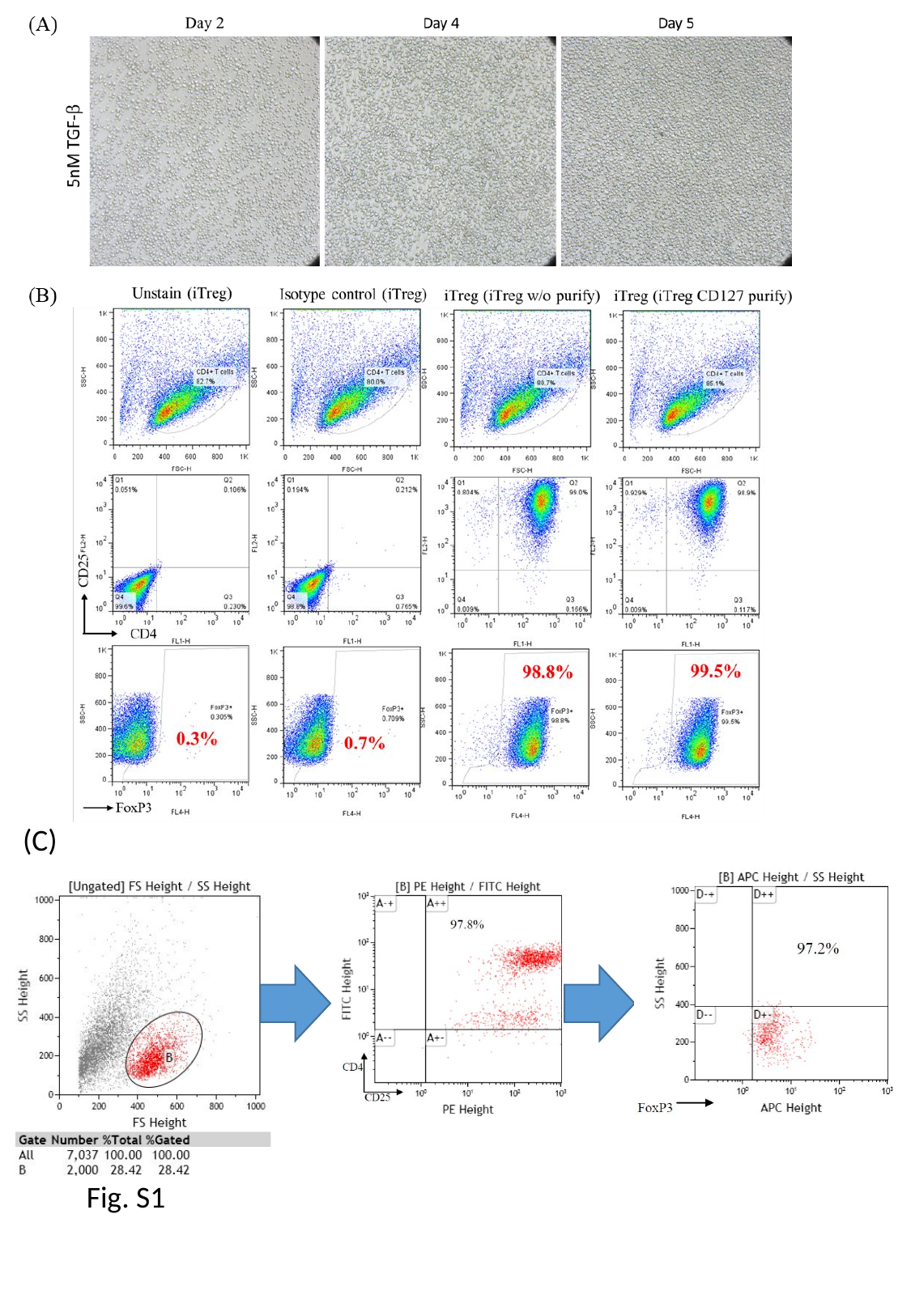

(C)
Fig. S1

Supplement: Supplementary file 1 — Supporting Information Figure S1: Shows the differentiation process of naïve CD4⁺ T cells into induced regulatory T cells (iTregs) and the analysis of Foxp3 expression by flow cytometry. [file JIMR-2026-4010824-s001.pptx]
